# Essential medicines for children: Should we focus on a priority list of medicines for the present?

**DOI:** 10.4103/0976-500X.77073

**Published:** 2011

**Authors:** B Gitanjali

**Affiliations:** *Section Editor, JPP*

The Better Medicines for Children (BMC) initiative of the World Health Organization (WHO) began in December 2007 as a consequence of the World Health Assembly resolution 60.20, when there was overwhelming evidence that nearly 50% of children under 5 years of age were dying of preventable diseases for which medicines existed. The need to improve access, develop “child friendly” formulations, conduct research, and use medicines in an optimal manner were the main objectives of the “make medicines child size” program. The initial global activity of this project was to prepare an Essential Medicines List for children (EMLc).[1] This was followed up with a children’s formulary,[2] identifying “child friendly” formulations and engaging health policy makers, decision makers, health care professionals, and representatives of professional associations in promoting the cause of BMC.

**Figure 1:**
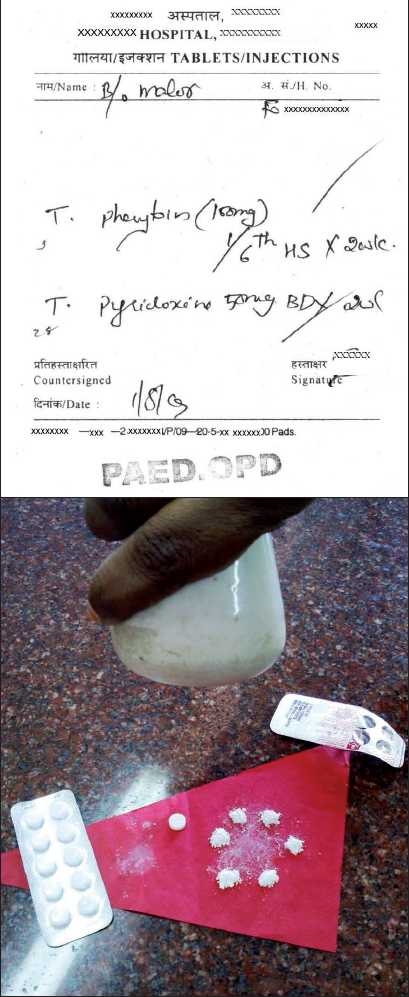
Prescription for phenytoin which will require the care-giver to break the tablet into six equal parts

The traditional method of treating children with oral medications in resource poor settings, is to fragment adult formulations and give it to them. If a child required 15 mg of phenytoin a day, the parent was given one tablet of 100 mg phenytoin and asked to divide it into six equal parts and give the required quanitity each day [Figure 1]. The arguments against procuring an oral liquid preparation of phenytoin are that it is expensive, its storage requires more room, the stability of the preparation in the extreme temperatures seen in our country are questionable, and there are no studies that show that this practice (of breaking tablets into small parts) produces poor clinical outcomes. This practice is by no means restricted to India and is seen in other resource-limited countries as well.

In Indonesia, the practice of preparing a “puyer” (Dutch term for powder) which is a powder containing three to four medicines all ground together is used commonly in pediatric medicine.[3] Therefore, children not only have to swallow very bitter medicines but the dose that they eventually swallow also may vary, depending on how careful the parent is in dividing the tablet and on how much a child may spit or vomit the medicine. This is a clear example of what was given by the Dutch to the country but remains in use when it has been long discarded by the Dutch in their country. Pharmacologically, children are now beginning to be recognized as a group with their own needs (and not as miniature adults) and at last there seems to be an acknowledgement of the need to address the issues relating to appropriate drug formulation for children, even in resource-limited settings.

Child friendly formulations are more expensive than the adult dosage counterparts, which is due mainly to the expense of formulations and also to some extent due to the packaging and a limited market. There is a limited demand because they are expensive which therefore leads to a smaller market. If procurement were to focus on child friendly formulations, a market and a demand will be created and there will be better availability. It is the creation of this critical mass that BMC hopes to bring about. India with its formidable sophisticated pharmaceutical capacity would be ideal for producing the formulations required for children, such as dispersible tablets. What is perhaps required is for the pediatricians and the health care policy makers to assure the industry that there is a firm commitment to prescribe and use these pediatric formulations, if they are produced.

The BMC project in India has its activities focused on two states, Chhattisgarh and Orissa. Under this project, Chhattisgarh has revised its EML, incorporating nearly 85% of the formulations mentioned in the WHO EMLc. This is the first state in India to specifically include pediatric formulations in its EML. Orissa has prepared a separate EMLc with 120 medicines. The Indian Academy of Pediatrics (IAP) has also developed an EMLc which is in the final stages of preparation. However, merely having children’s formulations on the lists is no guarantee that these medicines will be available in public health facilities. The gap between the EML and the procurement lists seems to be very wide, with most of the drugs falling through without being procured. In Chhattisgarh, public health facilities have only 17% availability of a limited list of essential medicines for children (Antony KR, personal communication), whereas in Orissa it is a mere 7% (Swain TR, personal communication).

A snapshot of the availability of five essential pediatric medicines in 126 public health facilities (data under communication) is a rude reminder that we have a long way to go. Oral rehydration salts (ORS) praised as one of the most important breakthroughs in the prevention of deaths due to diarrhea are not available in 10% of the health facilities. The DLHS-3 survey data indirectly confirm this finding of the snapshot survey as only one-third of children with diarrhea get ORS.[4] Six years after the IAP included zinc for the treatment of acute diarrhea in the consensus statement of its national task force,[5] it is still not available in two-thirds of the facilities (data under communication). The story is the same with vitamin A, with only 80% of the facilities having it. All these five surveyed medicines are commonly used, cheap, proven to improve childhood morbidity/mortality rates, included in the National Rural Health Mission list for subcenters, and widely manufactured in India, and yet they remain out of reach of the Indian child. If this is the case for just five essential medicines, what would be the case if the nearly 200 medicines in the WHO EMLc were to be included in the EML? How many will be procured even if they feature in the EMLc?

The WHO is looking at a limited list of 20 priority medicines for children, which are selected based on the evidence available to improve child survival as well as the fact that they are treatment options for the major causes of mortality and morbidity in children under 5 years of age. Medicines for the treatment of pneumonia, diarrhea, malaria, neonatal infections, HIV, tuberculosis, and palliative care as well as vitamin A (for improving child survival) will be the priority medicines which can easily be incorporated into any procurement list, even in resource poor settings. If this small number of medicines can be made available at public health facilities throughout the year and used by the health personnel, a significant impact on child morbidity and mortality can be expected.

If health is a fundamental right, should not every child in India have the right to essential medicines? It is sad that 35 years after the concept of essential medicines was propagated by the WHO, children are yet to benefit from its implementation. India, with its 400 million children, is the country with the largest number of children in the world. The BMC is a small beginning, but there can be no doubt that a successful campaign will not only affect the children of India but of the world too.
